# The Characteristics of Peripheral Blood Lymphocyte Subsets in HIV-related Diffuse Large B-cell Lymphoma Patients and Their Impact on Treatment Efficacy

**DOI:** 10.2174/011570162X369231250818043532

**Published:** 2025-08-26

**Authors:** Huiyu Xiang, Can Lin, Shuang Chen, Yu Peng, Tingting Jiang, Changhai Lin, Qing Xiao, Xiaomei Zhang, Tingting Liu, Nanjun Li, Xinyi Tang, Yakun Zhang, Junxi Liu, Zailin Yang

**Affiliations:** 1 School of Medicine, Chongqing University, Chongqing, 400030, China;; 2 Department of Hematology-Oncology, Chongqing Key Laboratory for the Mechanism and Intervention of Cancer Metastasis, Chongqing University Cancer Hospital, Chongqing, 400030, China;; 3 Department of Laboratory Medicine, Chongqing University Cancer Hospital, Chongqing, China;; 4 School of Life Sciences, Chongqing University, Chongqing, China

**Keywords:** HIV-associated lymphoma, diffuse large B-cell lymphoma, lymphocyte subset, antiretroviral therapy, treatment efficacy, CD4^+^ T-cells

## Abstract

**Introduction:**

Peripheral blood lymphocyte subsets have been shown to influence prognosis in HIV-associated Diffuse Large B-Cell Lymphoma (HIV-DLBCL), a rare and highly aggressive form of non-Hodgkin's lymphoma linked to immunosuppression and abnormal B-cell proliferation. To lay the foundation for individualized therapy based on factors such as CD4^+^/CD8^+^ ratio and Treg/NK cell characteristics, this retrospective study was conducted to explore the variations in lymphocyte subset levels.

**Methods:**

Overall, 51 HIV-DLBCL patients, 50 DLBCL patients, and 42 Healthy Donors (HD) were enrolled in the study. Data were extracted from outpatient records and the Hospital Information Management System. SPSS 27.0 software was used for statistical analysis of the data.

**Results:**

Significant differences in lymphocyte subsets were observed between groups. HIV-DLBCL patients showed decreased CD4^+^ T cell and regulatory T cell (Treg) counts/percentages compared to DLBCL patients and HD, but increased CD8^+^ T cell counts and percentages, as well as Treg percentages. Age-stratified analysis revealed that older HIV-DLBCL patients had lower CD8^+^ T cell counts, reduced CD3^+^ T cell percentages, and elevated CD56^+^CD16^+^ NK cell proportions compared to their younger counterparts.

**Discussion:**

This study revealed a distinct pattern of immune dysregulation in HIV-DLBCL patients, characterized by CD4^+^ T cell depletion and CD8^+^ T cell expansion, which is consistent with previous studies. Age-related immunosenescence may exacerbate the increased proportion of NK cells and the decline in T-cell function, suggesting a poorer prognosis in elderly patients. However, the lack of association between lymphocyte subsets and chemotherapy efficacy may reflect the broad impact of standard regimens on immune reconstitution. Limitations include the small sample size, absence of functional experiments, and failure to assess the influence of co-infections. Future studies should expand the cohort and integrate multi-omics data to validate these findings.

**Conclusion:**

Patients with HIV-DLBCL have distinctive alterations in peripheral blood lymphocyte subsets, such as a decreased absolute count and percentage of CD4^+^ T cells, in comparison to individuals with DLBCL. These alterations appear age-related and showed no significant association with prior antiretroviral therapy. The therapeutic effect of chemotherapy for HIV-DLBCL, however, might not be impacted by the low absolute count and percentage of CD4^+^ T-cells in peripheral blood, as well as whether or not they had previously received antiretroviral therapy.

## 1 INTRODUCTION

HIV-associated Diffuse Large B-Cell Lymphoma (HIV-DLBCL) is a rare, highly aggressive Non-Hodgkin Lymphoma (NHL) with incompletely understood pathogenesis. Immunosuppression and dysregulation of immune reconstitution are the primary etiology of HIV-DLBCL, which increases the risk of abnormal B-cell proliferation [1-4]. Compared with non-HIV-associated DLBCL, HIV-DLBCL is not only accompanied by a marked reduction of CD4^+^ T cells in peripheral blood and significant immunodeficiency, but also exhibits higher aggressiveness and heterogeneity [5-7]. Despite the remarkable success of Antiretroviral Therapy (ART) in improving survival and immune function in HIV-infected patients, the treatment of HIV-DLBCL patients remains suboptimal because of high relapse and mortality rates [5, 8-10].

While numerous studies have established the prognostic significance of peripheral blood lymphocyte subsets in the pathogenesis of HIV-DLBCL, critical knowledge gaps remain. Vaughan *et al*. [11] first demonstrated that monocyte fluorescence intensity, rather than absolute lymphocyte counts, better predicted survival in HIV-DLBCL patients, suggesting functional immune markers may be more clinically relevant than quantitative assessments alone. De Carvalho *et al*. [12] further characterized the unique molecular profile of HIV-DLBCL, revealing EBV-driven NF-κB activation that differentially impacts the distribution of lymphocyte subsets compared to HIV-negative DLBCL. Most importantly, Liévin *et al*. [13] recently identified that B-cell Activating Factor (BAFF) levels post-R-CHOP chemotherapy significantly modify the prognostic value of CD4^+^ T-cell counts—a finding particularly relevant to our study, as BAFF levels have never been examined in the context of ART-treated HIV-DLBCL patients. These collective findings highlight the complex interplay between viral factors, treatment effects, and immune cell populations that our study specifically addresses through comprehensive TBNK (T cells, B cells, and NK cells) and regulatory T cell (Treg) profiling. Furthermore, in the modern era of combination Antiretroviral Therapy (cART), HIV-DLBCL patients treated with R-CHOP are reported to have a 2-year overall survival rate of 75% and similar survival outcomes to DLBCL patients without HIV infection [14]. Lymphoid subset reduction and immunodeficiency are common after HIV infection, but this question, whether or not to receive ART therapy on HIV-DLBCL treatment outcomes and patient survival, is controversial, which is a concern for our study. As one of the most basic laboratory tests in HIV-infected patients, lymphocyte subsets in peripheral blood are important for monitoring impaired immune function in HIV-infected patients. However, the characteristics of peripheral blood lymphocyte subsets in HIV-DLBCL patients, differences in lymphocyte subsets between patients of different ages or treatment efficacy groups, as well as the impact of ART on the treatment outcomes of HIV-DLBCL, remain to be further explored.

In our study, we first compared the characteristics of TBNK and Treg among Healthy Donors (HD), DLBCL, and HIV-DLBCL patients. We subsequently explored differences in TBNK cell subsets and Tregs according to patient age or treatment response. Finally, we further analyzed the impact of Antiretroviral Therapy (ART) on treatment outcomes in HIV-DLBCL patients. Collectively, our findings help characterize lymphocyte subset profiles and clarify the relationship between ART and treatment response in HIV-DLBCL patients, providing new insights into the individualized treatment of HIV-DLBCL patients.

## 2 MATERIALS AND METHODS

### 2.1 Research Ethics Committee Approval

This study was approved by the Medical Ethics Committee of Chongqing University Cancer Hospital (CZLS2023085-A-100) and was conducted in accordance with the Declaration of Helsinki.

### 2.2 Research Design and Center

This retrospective study was conducted with newly diagnosed HIV-DLBCL and DLBCL patients from January 21, 2021 to June 25, 2024. Meanwhile, Healthy Donors (HD) who underwent physical examination during this period were selected as controls. Clinical and laboratory data of patients and healthy donors were obtained from outpatient medical records and Hospital Information Management System records.

Inclusion criteria were as follows: (1) Newly diagnosed HIV-associated Diffuse Large B-Cell Lymphoma (HIV-DLBCL) and DLBCL patients between January 21, 2021 and June 25, 2024, meeting the 2016 WHO Classification of Tumors of Hematopoietic and Lymphoid Tissues, (2) Healthy Donors (HD) who underwent physical examinations during the same period, and (3) Age >18 years. Exclusion criteria were as follows: (1) Patients with concurrent other malignancies or active autoimmune diseases, (2) Presence of severe infections (*e.g*., sepsis, active tuberculosis) or uncontrolled chronic diseases (*e.g*., hepatic or renal failure), and (3) Healthy controls with HIV positivity or other immunodeficiency conditions.

This study is a retrospective observational analysis utilizing only existing anonymized clinical data, involving no patient interventions or additional risks. Following review and approval by the Ethics Committee, the requirement for obtaining patient-informed consent was waived.

### 2.3 Patients

Overall, 51 patients with newly diagnosed HIV-DLBCL were enrolled in the study, including 39 males and 12 females, with ages ranging from 28 to 82 years old. 50 patients with DLBCL were enrolled, comprising 32 males and 18 females, with ages ranging from 30 to 88 years. All patients were diagnosed in accordance with the 2016 WHO Classification Criteria for Tumors of Hematopoietic and Lymphoid Tissues [15], and the efficacy of their treatments was determined based on the relevant criteria [16]. Additionally, 42 HD individuals who underwent routine physical examinations were enrolled as the control group, comprising 19 males and 23 females, aged between 20 and 78 years, with no signs of disease.

### 2.4 Clinical and Laboratory Information

The following key study characteristics were included in this study: age, gender, clinical diagnosis (DLBCL, HIV-DLBCL, HD), efficacy of the 4th course of chemotherapy (CR or CR not met), ART status at the initial diagnosis (ART-treated or ART-naive), blood routine examination (hemoglobin level, white blood cell counts, lymphocyte counts, lymphocyte percentage, platelet counts), lymphocyte subset indicators at the initial diagnosis and after four rounds of chemotherapy (counts and percentage of CD3^+^ T cell, CD8^+^ T cell, CD4^+^ T cell, CD19^+^ B cell, CD56^+^ CD16^+^ NK cell and Treg).

### 2.5 Instruments and Reagents

Flow cytometric analysis was performed using an EasyCell 206A1 flow cytometer (Shenzhen Wellgrow Technology Ltd., China). The following reagents were utilized: a six-color lymphocyte subset (TBNK) analysis kit, mouse anti-human monoclonal antibodies for regulatory T-cell (Treg) detection, and related supporting reagents (BEIJING TONGSHENG SHIDAI BIOTECH CO., LTD, China). The TBNK panel included fluorescently conjugated antibodies targeting CD3 FITC/CD16+56 PE/CD45 PerCP-Cy5.5/CD4 PE-Cy7/CD19 APC/CD8 APC-Cy7. For Treg cell identification, the following antibodies were employed: CD4 FITC, CD25 PE, CD3 PerCP, CD127 APC, and isotype controls (Mouse IgG1 FITC, PE, and APC).

### 2.6 Flow Cytometric Analysis

The absolute counts and percentages of peripheral blood TBNK and Treg populations were analyzed using flow cytometry. In brief, Peripheral blood samples (2 mL) anticoagulated with EDTA were first collected. Then, for TBNK subset quantification, 50 μL of whole blood was aliquoted into absolute counting tubes and stained with the pre-mixed TBNK antibody cocktail. For Treg analysis, 50 μL of whole blood was added to standard flow cytometry tubes containing the Treg-specific antibody mixture. Both tubes were incubated for 15 minutes at room temperature in the dark. Subsequently, 450 μL of lysing solution was added to each tube, followed by a 15-minute incubation for erythrocyte lysis. After lysis, TBNK samples were immediately analyzed on the flow cytometer. For Treg samples, the supernatant was discarded after centrifugation, and 500 µL of PBS was added before FCM analysis. Then, lymphocyte populations were gated using CD45 expression (horizontal axis) and side scatter (SSC, vertical axis). T cells (CD3+), B cells (CD19+), and NK cells (CD16+56+) were identified within the lymphocyte gate. For Treg analysis, CD3+CD4+ T cells were further gated to quantify CD4+CD25+CD127dim subsets. A minimum of 10,000 events per sample was acquired. Finally, data analysis was conducted using Kaluza software (Beckman Coulter).

### 2.7 Statistical Analysis

IBM SPSS 27.0 software was used to statistically analyze the data. In descriptive statistics, qualitative data are expressed as numbers and percentages; quantitative data that conform to a normal distribution are expressed as mean ± standard deviation (x ± s); and quantitative data that do not conform to a normal distribution are expressed as medians and quartiles (percentiles of 25% and 75%) (Median (Q1, Q3)). For comparisons between two groups of quantitative data, the independent samples t-test was used when the data conformed to normality and the variance was uniform. The t-test was used when the data conformed to normality but the variance was not uniform, and the Mann-Whitney U-test was used when the data belonged to a skewed distribution. For multiple independent samples, a one-way ANOVA was performed if the data from multiple samples were normally distributed and the variance aligned, and the Kruskal-Wallis test was used if they were skewed or the variance was not aligned. When analyzing the qualitative data of two dichotomous groups, the chi-square test was used when the total number of samples was greater than or equal to 40 and the expected frequency in each cell was ≥ 5, and the Fisher’s exact test was used if either of the above conditions was not met. *P*-value < 0.05 indicated that the difference was statistically significant. In this case, the Bonferroni method in SPSS was used to correct the test level for two-by-two comparisons between multiple samples.

## 3 RESULT

### 3.1 Comparison of Lymphocyte Subsets Among the HD, DLBCL, and HIV-DLBCL

A total of 51 HIV-DLBCL patients, 50 DLBCL patients, and 42 HD patients were enrolled in our study. The main characteristics of the included 3 groups of cases are summarized in Table **1**. Detailed subgroup analysis results can be found in Supplementary Table **S1**. Comparative analysis of lymphocyte subsets revealed significant alterations in HIV-DLBCL patients relative to DLBCL patients and HD. Specifically, HIV-DLBCL patients exhibited decreased counts of CD4^+^ T cells and Tregs, along with reduced CD4^+^ T cell percentage compared to DLBCL controls (*P* < 0.05), whereas CD8^+^ T cell counts, CD8^+^ T cell percentage, and Treg percentage were significantly elevated (*P* < 0.05). When compared to HD, the HIV-DLBCL cohort demonstrated further reductions in CD3^+^ T cells, CD4^+^ T cells, CD19^+^ B cells, CD56^+^CD16^+^ NK cells, and Treg counts, as well as diminished CD4^+^ T cell percentage (*P* < 0.05), concurrent with increased CD8^+^ T cell counts and elevated percentages of CD8^+^ T cells and Tregs (*P* < 0.05). Notably, DLBCL patients displayed a higher percentage of Tregs (*P* < 0.05) but lower counts of CD3^+^ T cells, CD8^+^ T cells, CD4^+^ T cells, CD19^+^ B cells, and CD56^+^CD16^+^ NK cells relative to HD (*P* < 0.05). However, no significant intergroup differences were observed in the percentages of CD3^+^ T cells, CD19^+^ B cells, or CD56^+^CD16^+^ NK cells across all cohorts.

### 3.2 Comparison of Lymphocyte Subsets in Different Age Groups

**Fig. (1):**
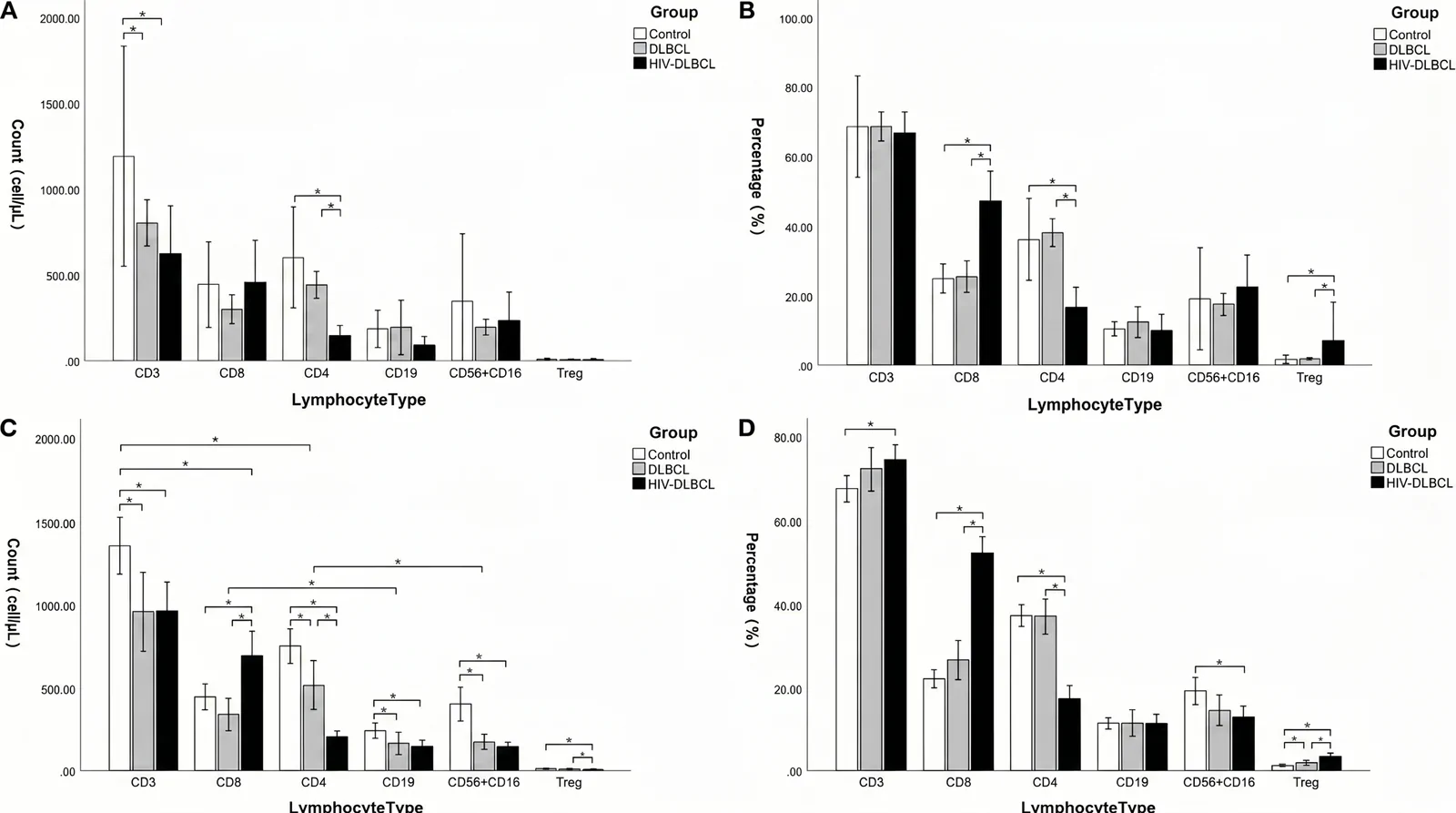
Histogram of lymphocyte subset levels in peripheral blood between HIV-DLCBL, DLCBL, and HD at different ages. (**A**) Comparison of lymphocyte counts in peripheral blood between HIV-DLCBL, DLCBL, and HD at age ≥ 60 years. (**B**) Comparison of lymphocyte percentages in peripheral blood between HIV-DLCBL, DLCBL, and HD at age ≥ 60 years. (**C**) Comparison of peripheral blood lymphocyte counts between HIV-DLCBL, DLCBL, and HD aged < 60 years. (**D**) Comparison of peripheral blood lymphocyte percentages between HIV-DLCBL, DLCBL, and HD aged < 60 years.

Given that advanced age (> 60 years) constitutes an International Prognostic Index (IPI) risk factor for DLBCL, we stratified patients by age (≥ 60 *vs*. < 60 years) to assess age-related variations in lymphocyte subsets. These age-related differences in lymphocyte subsets across HIV-DLBCL, DLBCL, and HD groups are detailed in Table **2**. Detailed subgroup analysis results can be found in Supplementary Table **S2**. Among HIV-DLBCL patients, those aged ≥ 60 years exhibited significantly elevated CD56^+^CD16^+^ NK cell percentage (*P* < 0.05) but reduced CD8^+^ T cell counts and CD3^+^ T cell percentage (*P* < 0.05) compared to younger patients, whereas no significant age-related differences emerged in DLBCL or HD groups. Subsequent comparative analyses revealed distinct patterns: In the ≥ 60-year cohort, HIV-DLBCL patients demonstrated higher CD8^+^ T cell and Treg percentages yet lower CD4^+^ T cell counts/percentage than DLBCL patients (*P* < 0.05), while also showing decreased CD3^+^ T cell and CD4^+^ T cell counts, diminished CD4^+^ T cell percentage, as well as elevated CD8^+^ T cell and Treg percentages relative to HD (*P* < 0.05); DLBCL patients concurrently displayed reduced CD3^+^ T cell counts *versus* HD (Fig. **1A**, **B**). Among <60-year patients, HIV-DLBCL cases exhibited increased CD8^+^ T cell counts/percentage and Treg percentage but decreased CD4^+^ T cell counts/ percentage and Treg counts compared to DLBCL patients (*P* < 0.05), while demonstrating broad reductions in percentages of CD3^+^ T cells and CD4^+^ T cells, as well as counts of CD3^+^ T cells, CD4^+^ T cells, CD19^+^ B cells, CD56^+^CD16^+^ NK cells, and Tregs *versus* HD (*P* < 0.05), despite elevated CD8^+^ T cell counts/percentage, CD56^+^CD16^+^ NK cell and Treg percentages (*P* < 0.05). Moreover, DLBCL patients consistently showed lower counts of CD3^+^ T cells, CD4^+^ T cells, CD19^+^ B cells, and CD56^+^CD16^+^ NK cells with a higher percentage of Tregs than HD (*P* < 0.05) (Fig. **1C**, **D**).

### 3.3 Comparison of Lymphocyte Subsets in Patients Achieved Different Therapeutic Effects of Chemotherapy

**Fig. (2):**
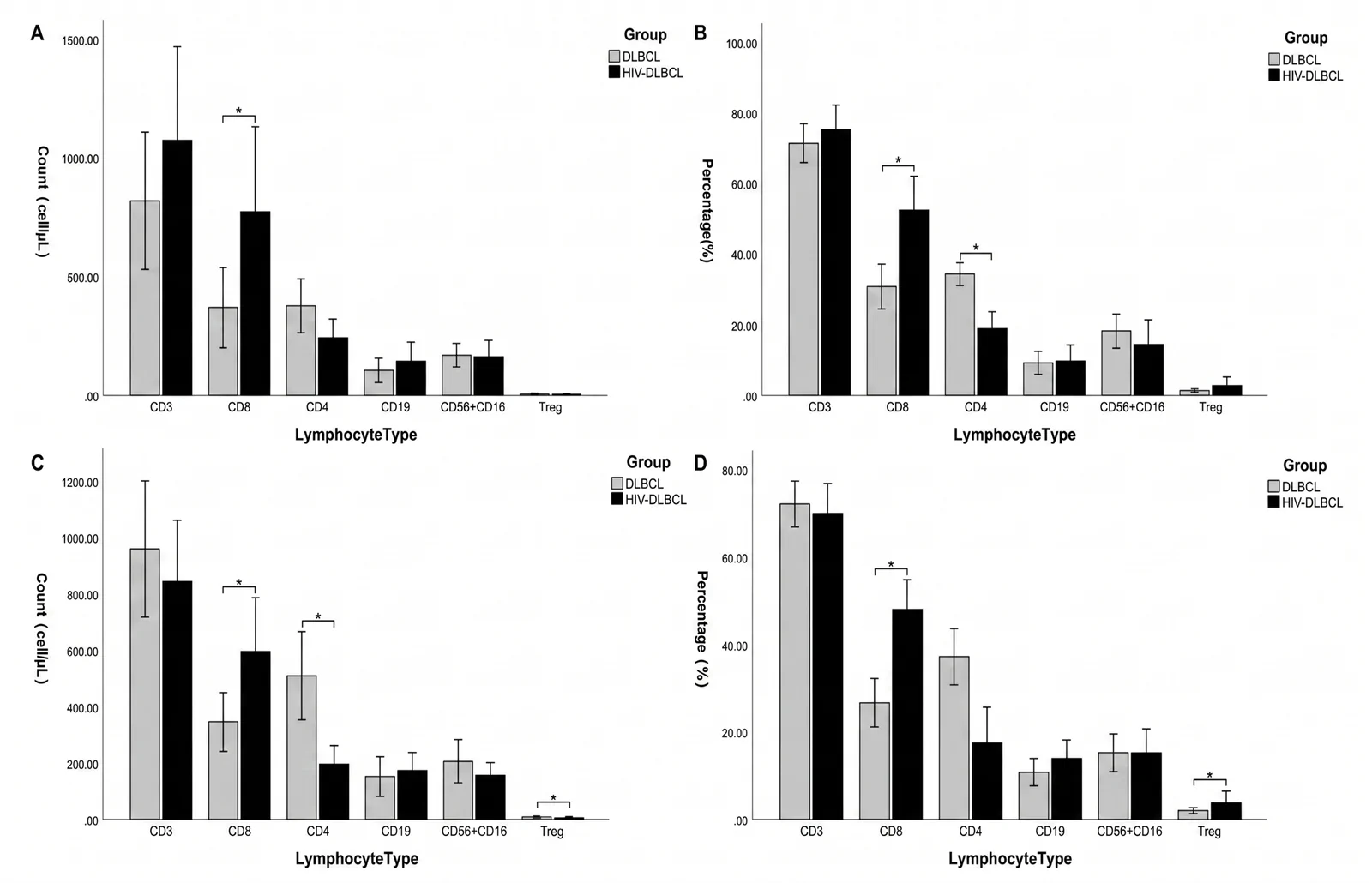
Histogram of peripheral blood lymphocyte subset levels in patients who achieved different therapeutic effects of chemotherapy. (**A**) Comparison of peripheral blood lymphocyte counts in patients who achieved CR (HIV-DLBCL *versus* DLBCL). (**B**) Comparison of peripheral blood lymphocyte percentage in patients who achieved CR (HIV-DLBCL *versus* DLBCL). (**C**) Comparison of peripheral blood lymphocyte counts in patients who had not achieved CR (HIV-DLBCL *versus* DLBCL). (**D**) Comparison of peripheral blood lymphocyte percentage in patients who had not achieved CR (HIV-DLBCL versus DLBCL).

To determine whether chemotherapy response influences lymphocyte profiles, we analyzed flow cytometry data of lymphocyte subsets from 27 HIV-DLBCL and 32 DLBCL patients after four chemotherapy cycles. As shown in Table **3**, a comparative assessment revealed no significant differences in peripheral blood lymphocyte levels between patients who achieved a Complete Response (CR; n = 11) and those who did not (CR not met; n = 16) within the HIV-DLBCL cohort. Similarly, DLBCL patients exhibited comparable lymphocyte distributions irrespective of CR status. Detailed subgroup analysis results can be found in Supplementary Table **S3**. Particularly, a substantially lower CD4^+^ T cell percentage and a significantly higher CD8^+^ T cell count/percentage were found in HIV-DLBCL patients who achieved CR (*P* < 0.05) (Fig. **2A**, **B**). As for the patients who had not achieved CR, significantly higher CD8 T^+^ cell counts/percentage and Treg percentage, while lower counts of CD4^+^ T cells and Treg were confirmed in HIV-DLBCL patients (Fig. **2C**, **D**).

### 3.4 Comparison of Lymphocyte Subsets and Treatment Efficacy in HIV-DLBCL Patients Receiving or Not Receiving ART

Given the potential impact of ART on immune recovery, we further compared the lymphocyte subsets and treatment response between ART-treated and ART-naive HIV-DLBCL patients to assess the effect of ART on these outcomes. Among the 51 HIV-DLBCL patients analyzed, 32 had received ART at the initial diagnosis, while the remaining 19 were ART-naive. Comparative analysis revealed no significant differences in lymphocyte subset profiles between ART-treated and ART-naive patients (Table **4**). Detailed subgroup analysis results can be found in Supplementary Table **S4**. Of the 27 HIV-DLBCL patients who completed four chemotherapy cycles with efficacy assessments, 19 had received ART. Subsequent evaluation demonstrated no association between ART exposure status and CR achievement (Table **5**).

## 4 DISCUSSION

The survival of HIV-infected individuals has significantly improved with widespread Antiretroviral Therapy (ART), yet the incidence of lymphoma continues to rise [17-20]. HIV infection disrupts immune homeostasis and lymphocyte function [21], notably impairing B-cell maturation and skewing the balance of lymphocyte subsets [22, 23]. Patients with low CD4^+^ T cell counts face a particularly elevated risk of developing lymphomas [24-27], with DLBCL being the most prevalent subtype in HIV cohorts. Although ART has significantly improved the prognosis of HIV patients, those with HIV-DLBCL still face high relapse rates and mortality [5, 8-10]. Thus, characterizing peripheral blood lymphocyte subset alterations and their clinical relevance in HIV-DLBCL may inform novel diagnostic and prognostic strategies.

In our study, we first analyzed TBNK and Treg cell profiles in HIV-DLBCL patients, DLBCL patients, and healthy donors. Our results shows that HIV-DLBCL patients exhibited significantly depleted CD4^+^ T cell and Treg counts alongside reduced CD4^+^ T cell proportions compared to DLBCL patients, while demonstrating broad lymphopenia (CD3^+^/CD4^+^ T cells, CD19^+^ B cells, CD56^+^CD16^+^ NK cells, Tregs) relative to healthy donors, which consistent with prior reports of impaired cellular immunity in HIV-DLBCL [12]. HIV-induced CD4^+^ T cell depletion promotes lymphomagenesis *via* multiple mechanisms. Chronic HIV infection drives Treg expansion *via* TGF-β/IL-10 signaling [28], while these Tregs exhibit elevated PD-L1 expression in HIV^+^ individuals [29], potentially explaining the impaired effector T cell function we observed. Intriguingly, Nie *et al*. [30] recently demonstrated that tumor-infiltrating Tregs in HIV-DLBCL patients may enhance chemosensitivity when ART is maintained—a critical context for interpreting our findings regarding treatment efficacy. Therefore, it makes it harder for immune systems to identify and eliminate tumor cells in HIV-DLBCL patients, which raises the risk of cancer and disease recurrence. Notably, HIV-DLBCL patients had higher CD8^+^ T cell counts and percentages of CD8^+^ T cells and Treg than DLBCL patients or HD, consistent with studies by Nie P *et al*. [30] and Wang XM *et al*. [31]. Long-term HIV-driven immunological activation promotes CD8^+^ T cell proliferation, but this prolonged stimulation may lead to T cell exhaustion and reduced antitumor capacity [32, 33]. Elevated Treg percentages in HIV-infected individuals likely reflect compensatory responses to chronic inflammation [34-36], yet they may also suppress anti-tumor immunity by inhibiting effector T cell function [37, 38].

Considering that advanced age (> 60 years) is a recognized risk factor in the IPI prognostic scoring system for DLBCL, we then categorized patients into two age groups to evaluate age-dependent differences in lymphocyte subset profiles. Our results revealed that HIV-DLBCL patients aged ≥ 60 years exhibited significantly higher percentages of CD56^+^CD16^+^ NK cells but lower CD8^+^ T cell counts and CD3^+^ T cell percentages compared to those < 60 years. This aligns with Deeks *et al*.'s findings [39], suggesting that immunosenescence contributes to these disparities. Aging-related immune decline in older HIV-DLBCL patients is characterized by increased NK cell proportions and reduced T cell quantity/function [40, 41]. These age-dependent immune alterations may exacerbate immunosuppression in HIV-DLBCL, potentially impacting disease prognosis and progression [42, 43].

We next analyzed lymphocyte subset variations among patients with different treatment responses. No significant differences in lymphocyte levels were found between HIV-DLBCL patients who achieved CR and those who did not, consistent with Huguet *et al*. [44]. This may be attributed to comparable immunomodulatory effects of standard regimens (R-CHOP/R-EPOCH) on lymphocyte counts across patients, as documented in prior studies [13, 44].

Considering the dual challenges of ART in HIV-DLBCL patients, including the risk of Immune Reconstitution Inflammatory Syndrome (IRIS) and potential drug interactions with chemotherapy, we sought to systematically evaluate the immunomodulatory effects of ART independent of other variables. Our analysis revealed no significant differences in peripheral blood lymphocyte subset levels between ART-treated and ART-naive HIV-DLBCL patients. This finding may be attributed to the prolonged immune reconstitution window required for ART, as evidenced by prior studies showing that the timing of ART initiation profoundly influences CD4/CD8 ratio restoration [45-47]. For instance, only patients initiating ART during the acute HIV infection phase (within 4 months) achieved CD4/CD8 normalization within 6-10 years, whereas chronic-phase starters failed to restore normal ratios even after 14 years of therapy. Thus, the insufficient recovery duration for immune reconstitution in our cohort likely underlies the lack of discernible lymphocyte subset differences between ART groups.

The lymphocyte profile identified in this study has the potential to be translated into clinical markers. The CD8+/Treg ratio was significantly elevated in non-CR patients aged ≥ 60 years and may serve as a simple prognostic scoring indicator, preferable to the age-alone component of the traditional IPI score [48]. Elderly patients with > 30% decline in CD56^+^CD16^+^ NK cells after chemotherapy have significantly lower 2-year PFS rates [41], which could then be included in efficacy monitoring programs.

While this study delineates the evolutionary characteristics and clinical significance of lymphocyte subsets in HIV-DLBCL patients through multi-group comparisons, certain limitations remain. First, its retrospective design and small sample size may compromise statistical robustness. Prospective studies with larger cohorts are needed to validate these findings. Second, due to the absence of immune function assays conducted at our institution, such data are lacking. Given that immune function influences disease progression and treatment response, future analyses of functional immunity will enhance the reliability of relevant studies. For example, Wang *et al*. [31] demonstrated that HIV-infected patients exhibit significant reductions in peripheral blood CD4^+^ and CD8^+^ naive T cells, accompanied by increased frequencies of highly cytotoxic CD4^+^/CD8^+^ T cell subsets and clonally expanded T cells. Notably, ART treatment was associated with a decrease in effector T cell counts and a gradual recovery of naive T cell populations. Third, the impact of co-infections on lymphocyte subsets was not evaluated. HIV-infected patients are prone to opportunistic infections, which may independently alter immune profiles and confound DLBCL outcomes [13, 49]. Future research should incorporate co-infection status to clarify this relationship. Fourth, although ART use was documented, treatment duration and medication adherence were not assessed. Short-term (< 6 months) *versus* long-term (> 2 years) ART may have opposite effects on immune reconstitution [29]. 30-50% of HIV patients were found to have missed doses [26], potentially confounding efficacy analyses. Lastly, clinical prognostic factors, such as disease stage, performance status, and symptom burden [48, 50], were not integrated with lymphocyte subset data. Multivariate analyses in future studies will better define how subset alterations interact with standard prognostic markers to influence HIV-DLBCL outcomes.

Taken together, this study offers fresh perspectives on the immunological profile of HIV-DLBCL patients. The improvement of the above shortcomings will contribute to further achievement in this field and provide a more accurate clinical foundation for treatment.

## CONCLUSION

Our study highlights distinct alterations in lymphocyte subsets among HIV-DLBCL patients, emphasizing their potential clinical and prognostic relevance. The observed reductions in CD4^+^ T cell and Treg counts, accompanied by increased CD8^+^ T cell and Treg percentages, suggest a profound immune dysregulation that distinguishes HIV-DLBCL from DLBCL and healthy individuals. These alterations were particularly pronounced in elderly patients, indicating an age-associated immunosenescence pattern, which may contribute to poorer immune surveillance and disease progression. Despite prior antiretroviral therapy, lymphocyte subset profiles remained altered, underscoring the complex interplay between HIV infection, lymphoma biology, and immune restoration. Importantly, we found no clear association between lymphocyte subset levels and treatment response, implying that standard immunophenotyping may have limited predictive value for short-term chemotherapy efficacy. However, markers such as an elevated CD8^+^/Treg ratio in elderly non-responders may offer future prognostic utility.

## Figures and Tables

**Table 1:** Baseline demographics and disease characteristics (Median (Q1, Q3)).

**-**	**HIV-DLBCL**	**DLBCL**	**HD**	***P*-value**
	**n=51**	**n=50**	**n=42**	
Gender	-	-	-	**0.008**
Female	12 (23.53%)	18 (36.00%)	23 (54.76%)	-
Male	39 (76.47%)	32 (64.00%)	19 (45.24%)	-
Age (years)	-	-	-	**<0.001**
<60	40 (78.43%)	20 (40.00%)	37 (88.10%)	-
≥60	11 (21.57%)	30 (60.00%)	5 (11.9%)	-
CBC	-	-	-	-
Hb (cells/μL)	**115.00(99.00,131.00)^#^**	**123.00(109.75,134.00)^#^**	143.00(131.75,156.00)	**<0.001**
WBC (cells/μL)	4.75(3.62,6.26)^*#^	6.30(4.86,7.44)	5.44(4.79,6.58)	**<0.001**
Lym %	24.30(16.40,32.60)^*#^	**20.30(14.65,25.33)^#^**	31.05(26.30,39.43)	**<0.001**
Lym (cells/μL)	**1.11(0.74,1.56)^#^**	**1.18(0.91,1.47)^#^**	1.87(1.41,2.28)	**<0.001**
PLT (cells/μL)	195.00(148.00,282.00)	240.50(177.50,302.25)	228.50(187.75,261.00)	0.111
TBNK and Treg	-	-	-	-
CD3^+^(cells/μL)	**749.00(543.00,1178.00)^#^**	**760.00(612.50,1094.75)^#^**	1235.50(1035.75,1621.25)	**<0.001**
CD8^+^(cells/μL)	565.00(294.00,821.00)^#*^	**243.00(162.50,401.75)^#^**	419.50(305.00,564.50)	**<0.001**
CD4^+^(cells/μL)	182.00(107.00,261.00)^#*^	**434.00(305.50,566.50)^#^**	691.50(524.75,897.25)	**<0.001**
CD19 (cells/μL)	**123.00(45.00,217.00)^#^**	**120.50(46.75,194.75)^#^**	209.00(132.75,314.75)	**<0.001**
CD56^+^ CD16^+ ^(cells/μL)	**133.00(81.00,217.00)^#^**	**156.50(92.00,265.75)^#^**	290.50(181.25,521.00)	**<0.001**
Treg (cells/μL)	4.50(2.00,7.25)^#*^	8.00(4.00,11.00)	8.00(3.75,14.00)	**0.013**
CD3^+^ %	74.50(64.36,80.95)	69.77(62.78,76.36)	68.36(63.04,75.07)	0.073
CD8^+^ %	52.41(41.92,59.50)^#*^	23.34(17.76,32.76)	22.78(16.92,28.22)	**<0.001**
CD4^+^ %	15.47(11.20,22.16)^#*^	36.94(32.41,43.22)	37.78(30.10,43.44)	**<0.001**
CD19^+^ %	10.23(5.90,15.70)	12.23(4.53,15.98)	10.58(8.64,13.28)	0.822
CD56^+^CD16^+^ %	10.50(7.12,22.34)	15.41(8.8,25.04)	16.66(12.05,24.72)	0.072
Treg%	3.19(1.98,4.49)^#*^	1.68(1.09,2.26)	1.14(0.63,1.88)	**<0.001**

**Note:** HIV-DLBCL, HIV-associated diffuse large B-cell lymphoma; HD, healthy donors; CBC, complete blood count; Hb, hemoglobin; Lym, lymphocyte; PLT, blood platelet; TBNK, T cells, B cells, and NK cells; Treg, regulatory T cells. The Shapiro-Wilk normality test and Brown-Forsythe test were performed to test normality and homogeneity of variance. The t-test or Mann-Whitney U test was used to compare differences between two groups and determine *P* values. The one-way ANOVA or Kruskal-Wallis test was used to compare differences among three or more groups and determine *P* values. The bold values represent *p*<0.05, indicating statistical significance.

^*^ *P* < 0.05 *vs*. DLBCL group; ^#^ *P* < 0.05 *vs*. control group.

**Table 2:** Comparison of peripheral blood lymphocyte subset levels in DLBCL, HIV-DLBCL, and HD at various ages (Median (Q1, Q3)).

**Groups**	**HIV-DLBCL (n=51)**		**DLBCL (n=50)**		**HD (n=42)**	
	**≥ 60 Years** **(n=11)**	**<60 Years** **(n=40)**	**≥ 60 Years (n=30)**	**<60 Years** **(n=20)**	**≥ 60 Years** **(n=5)**	**<60 Years** **(n=37)**
CD3^+^ (cells/μL)	543.00 (304.00,746.00)	830.00 (664.00,1231.25)	724.00 (599.75,974.00)	816.00 (618.25,1204.00)	**1127.90** (699.33,1710.59 )^*#^	**1250.53** **(1049.61,1621.68)^$&^**
CD8^+^ (cells/μL)	440.00 (157.00,596.00)	**585.00** (415.50,909.75)^*^	215.00 (151.75,386.25)	**268.00** **(169.75,461.00)^$^**	491.46 (231.79,631.62)	**418.22** **(311.41,542.40)^$^**
CD4^+^ (cells/μL)	146.00 (.49.00,191.00)	193.00 (111.25,270.25)	**406.50** (311.25,505.25)^*^	**488.00** **(289.50,769.25)^$^**	**525.20** (422.48,817.43)^*^	**695.84** **(526.28,897.31)^$&^**
CD19^+^ (cells/μL)	49.00 (28.00,159.00)	129.00 (47.50,248.25)	103.50 (39.25,164.25)	132.50 (50.00,256.00)	216.85 (96.24,257.01)	**209.01** **(136.35,327.84)^$&^**
CD56^+^ CD16^+^ (cells/μL)	136.00 (91.00,274.00)	131.50 (80.25,204.75)	156.50 (91.75,265.75)	157.50 (102.00,288.50)	175.87 (169.68,603.55)	**339.74** **(194.60,546.67)^$&^**
Treg (cells/μL)	4.53 (1.19,7.78)	4.18 (2.40,7.48)	6.00 (4.00,10.25)	**9.00** **(4.25,12.50)^$^**	8.10 (2.82,11.95)	**8.04** **(3.68,14.39)^$^**
CD3^+^ %	65.38 (63.60,74.50)	**75.80** (65.32,82.00)^*^	68.25 (60.75,74.88)	74.77 (64.20,78.74)	71.38 (59.30,76.57)	**67.88** **(62.83,75.22)^$^**
CD8^+^ %	52.84 (34.40,57.89)	52.36 (43.98,61.13)	**23.31** **(16.63,31.39)**	**23.34** **(19.33,33.01)^$^**	**24.58** (21.67,28.34)^*^	**22.65** **(16.61,28.22)^$^**
CD4^+^ %	14.50 (8.51,23.00)	15.49 (12.16,21.47)	**36.31** (31.41,41.51)^*^	**38.07** **(33.54,43.97)^$^**	**40.80** (26.30,43.65)^*^	**37.42** **(30.32,43.53)^$^**
CD19^+^ %	9.93 (5.77,15.79)	10.54 (6.46,15.59)	10.31 (4.53,16.37)	13.22 (4.08,15.88)	10.10 (8.95,817.43)	10.83 (8.58,14.05)
CD56^+^ CD16^+^ %	22.34 (9.30,31.00)	**9.39** (6.89,17.77)^*^	18.65 (8.93,25.35)	12.99 (7.73,22.90)	16.41 (10.67,28.85)	**16.91** **(11.75,24.74)^$^**
Treg%	3.28 (2.24,13.66)	3.18 (1.92,4.53)	**1.77** **(0.96,2.29)**	**1.50** **(1.33,2.30)^$^**	**1.75** (0.44,2.44)^*^	**1.14** **(0.63,1.82)^$&^**

**Note:** HIV-DLBCL, HIV-associated diffuse large B-cell lymphoma; HD, healthy donors; Treg, regulatory T cells. The Shapiro-Wilk normality test and Brown-Forsythe test were performed to test normality and homogeneity of variance. The t-test or Mann-Whitney U test was used to compare differences between two groups and determine *P* values. The one-way ANOVA or Kruskal-Wallis test was used to compare differences among three or more groups and determine *P* values. The bold values represent *p*<0.05, indicating statistical significance.

^*^ *P*<0.05 *vs*. the group of HIV-related DLBCL patients aged ≥ 60 years.

^#^ *P*<0.05 *vs*. the group of DLBCL patients aged ≥ 60 years.

^$^ *P*<0.05 *vs*. the group of HIV-related DLBCL patients aged < 60 years.

^&^ *P*<0.05 *vs*. the group of DLBCL patients aged < 60 years.

**Table 3:** Comparison of peripheral blood lymphocyte subset levels across different treatment response groups after four cycles of chemotherapy. (Median (Q1, Q3)).

**Groups**	**HIV-DLBCL (n=27)**		**DLBCL (n=32)**	
	**Achievement of CR (n=11)**	**CR Not Met (n=16)**	**Achievement of CR (n=15)**	**CR Not Met (n=17)**
CD3^+^ (cells/μL)	1117.00(746.00,1241.00)	762.50(546.75,1093.00)	718.00(321.00,1144.00)	801.00(658.50,1143.00)
CD8^+^ (cells/μL)	725.00(513.00,810.00)	527.00(324.00,813.25)	219.00(154.00,509.00)^*^	**269.00(182.00,556.50)^#^**
CD4^+^ (cells/μL)	244.00(146.00,360.00)	188.50(101.00,266.25)	379.00(187.00,506.00)	**391.00(302.00,714.50)^#^**
CD19^+^ (cells/μL)	159.00(46.00,184.00)	153.50(56.25,291.00)	95.00(12.00,129.00)	134.00(51.00,183.50)
CD56^+^ CD16^+^ (cells/μL)	134.00(91.00,238.00)	140.50(95.00,231.50)	127.00(88.00,258.00)	182.00(96.50,321.00)
Treg (cells/μL)	4.35(3.30,5.62)	2.80(1.36,4.00)	4.00(2.00,8.00)	**9.00(4.00,12.00)^#^**
CD3^+^ %	76.33(63.40,80.61)	68.93(61.73,78.22)	70.22(64.33,79.47)	73.04(62.47,78.77)
CD8^+^ %	49.81(41.92,59.50)	48.59(37.20,60.14)	29.89(18.03,35.89)^*^	**23.84(21.34,34.68)^#^**
CD4^+^ %	19.13(14.50,25.20)	14.53(8.77,22.10)	35.13(29.78,38.66)^*^	36.78(29.76,43.47)
CD19^+^ %	10.80(3.42,15.11)	13.90(10.60,17.18)	8.13(4.62,15.01)	12.57(4.76,15.64)
CD56^+^ CD16^+^ %	9.30(7.50,25.30)	15.24(6.50,21.90)	19.62(8.88,25.62)	17.39(6.71,23.00)
Treg%	2.88(1.30,4.14)	2.78(2.05,5.03)	1.62(0.80,2.10)	**1.72(1.21,2.90)^#^**

**Note:** HIV-DLBCL, HIV-associated diffuse large B-cell lymphoma; Complete Response (CR); Treg, regulatory T cells. The Shapiro-Wilk normality test and Brown-Forsythe test were performed to test normality and homogeneity of variance. The t-test or Mann-Whitney U test was used to compare differences between two groups and determine *P *values. The one-way ANOVA or Kruskal-Wallis test was used to compare differences among three or more groups and determine *P* values. The bold values represent *p*<0.05, indicating statistical significance.

^*^ *P* < 0.05 *vs*. the group of HIV-DLBCL patients who achieved CR.

^#^ *P* < 0.05 *vs*. the group of HIV-DLBCL patients who did not achieve CR.

**Table 4:** Comparison of peripheral blood lymphocyte subsets between ART-treated and ART-naive HIV-DLBCL patients (Median (Q1, Q3)).

**Groups**	**ART-treated Group (n=32)**	**ART-naive Group (n=19)**	***P*-value**
CD3^+^ (cells/μL)	1117.00(746.00,1241.00)	762.50(546.75,1093.00)	0.276
CD8^+^ (cells/μL)	725.00(513.00,810.00)	527.00(324.00,813.25)	0.397
CD4^+^ (cells/μL)	244.00(146.00,360.00)	188.50(101.00,266.25)	0.101
CD19^+^ (cells/μL)	159.00(46.00,184.00)	153.50(56.25,291.00)	0.091
CD56^+^ CD16^+^ (cells/μL)	134.00(91.00,238.00)	140.50(95.00,231.50)	0.134
Treg (cells/μL)	4.35(3.30,5.62)	2.80(1.36,4.00)	0.061
CD3^+^ %	76.33(63.40,80.61)	68.93(61.73,78.22)	0.825
CD8^+^ %	49.81(41.92,59.50)	48.59(37.20,60.14)	0.224
CD4^+^ %	19.13(14.50,25.20)	14.53(8.77,22.10)	0.154
CD19^+^ %	10.80(3.42,15.11)	13.90(10.60,17.18)	0.121
CD56^+^ CD16^+^ %	9.30(7.50,25.30)	15.24(6.50,21.90)	0.403
Treg%	2.88(1.30,4.14)	2.78(2.05,5.03)	0.262

**Note:** HIV-DLBCL, HIV-associated diffuse large B-cell lymphoma; ART-treated, HIV-DLBCL patients who have received antiretroviral treatment at the initial diagnosis; ART-naive, HIV-DLBCL patients who have not received antiretroviral treatment at the initial diagnosis; Treg, regulatory T cells. The Shapiro-Wilk normality test and Brown-Forsythe test were performed to test normality and homogeneity of variance. The t-test or Mann-Whitney U test was used to compare differences between two groups and determine P values. The one-way ANOVA or Kruskal-Wallis test was used to compare differences among three or more groups and determine *P* values. *P* < 0.05 means statistically significant.

**Table 5:** Impact of ART treatment exposure on the treatment response of HIV-DLBCL patients.

**ART Status**	**Treatment Response**		***P*-value**
	**Achievement of CR** **(n=11)**	**CR Not Met** **(n=16)**	
ART-treated	7 (36.8%)	12 (63.2%)	0.525
ART-naive	4 (50.0%)	4 (50.0%)	

**Note:** HIV-DLBCL, HIV-associated diffuse large B-cell lymphoma; ART-treated, HIV-DLBCL patients who have received antiretroviral treatment at the initial diagnosis; ART-naive, HIV-DLBCL patients who have not received antiretroviral treatment at the initial diagnosis; Complete Response (CR).

## Data Availability

All data generated or analyzed during this study are included in this published article.

## LIST OF ABBREVIATIONS

APC: Allophycocyanin
ART: Antiretroviral Therapy
BAFF: B-cell Activating Factor
cART: Combination Antiretroviral Therapy
CBC: Complete Blood Count
CR: Complete Response
DLBCL: Diffuse Large B-cell Lymphoma
EBV: Epstein-Barr Virus
EDTA: Ethylenediaminetetraacetic Acid
FCM: Flow Cytometry
FITC: Fluorescein Isothiocyanate
Hb: Hemoglobin
HD: Healthy Donors
HIV: Human Immunodeficiency Virus
HIV-DLBCL: HIV-associated Diffuse Large B-cell Lymphoma
IgG: Immunoglobulin G
IPI: International Prognostic Index
IRIS: Immune Reconstitution Inflammatory Syndrome
Lym: Lymphocyte
NK cells: Natural Killer cells
PBS: Phosphate-Buffered Saline
PE: Phycoerythrin
PerCP: Peridinin-Chlorophyll-Protein
PFS: Progression-Free Survival
PLT: Platelet
R-CHOP: Rituximab, Cyclophosphamide, Doxorubicin, Vincristine, Prednisone
R-EPOCH: Rituximab, Etoposide, Prednisone, Vincristine, Cyclophosphamide, Doxorubicin
SSC: Side Scatter
TBNK: T cells, B cells, and NK cells
Treg: Regulatory T cells
WBC: White Blood Cell

## STANDARDS OF REPORTING

STROBE guidelines were followed.

## References

[1] Dolcetti R., Gloghini A., Caruso A., Carbone A. (2016). A lymphomagenic role for HIV beyond immune suppression?. Blood.

[2] Bibas M., Antinori A. (2009). EBV and HIV-related lymphoma.. Mediterr. J. Hematol. Infect. Dis..

[3] Cesarman E. (2013). Pathology of lymphoma in HIV.. Curr. Opin. Oncol..

[4] Shiels M.S., Engels E.A. (2017). Evolving epidemiology of HIV-associated malignancies.. Curr. Opin. HIV AIDS.

[5] Wang C., Xiao Q., Zhang X., Liu Y. (2024). HIV associated lymphoma: Latest updates from 2023 ASH annual meeting.. Exp. Hematol. Oncol..

[6] Zhou M., Cheng J., Zhao H. (2022). Clinical features, phenotypic markers and outcomes of diffuse large b-cell lymphoma between hiv-infected and hiv-uninfected chinese patients.. Cancers.

[7] Liu Y., Xie X., Li J. (2024). Immune characteristics and immunotherapy of hiv-associated lymphoma.. Curr. Issues Mol. Biol..

[8] Manosuthi W., Charoenpong L., Santiwarangkana C. (2021). A retrospective study of survival and risk factors for mortality among people living with HIV who received antiretroviral treatment in a resource-limited setting.. AIDS Res. Ther..

[9] Schwetz T.A., Fauci A.S. (2019). The extended impact of human immunodeficiency virus/aids research.. J. Infect. Dis..

[10] Berhan A., Bayleyegn B., Getaneh Z. (2022). HIV/AIDS associated lymphoma: Review.. Blood Lymphat. Cancer.

[11] Vaughan J., Wiggill T., Lawrie D., Machaba M., Patel M. (2023). The prognostic impact of monocyte fluorescence, immunosuppressive monocytes and peripheral blood immune cell numbers in HIV-associated Diffuse Large B-cell Lymphoma.. PLoS One.

[12] de Carvalho P.S., Leal F.E., Soares M.A. (2021). Clinical and molecular properties of human immunodeficiency virus-related diffuse large B-cell lymphoma.. Front. Oncol..

[13] Liévin R., Maillard A., Hendel-Chavez H. (2024). Immune reconstitution and evolution of B-cell–stimulating cytokines after R-CHOP therapy for HIV-associated DLBCL.. Blood Adv..

[14] Allred J., Bharucha K., Özütemiz C. (2021). Chimeric antigen receptor T-cell therapy for HIV-associated diffuse large B-cell lymphoma: Case report and management recommendations.. Bone Marrow Transplant..

[15] Arber D.A., Orazi A., Hasserjian R. (2016). The 2016 revision to the World Health Organization classification of myeloid neoplasms and acute leukemia.. Blood.

[16] (2022). Health Commission of the PRC N. National guidelines for diagnosis and treatment of malignant lymphoma 2022 in China (English version).. Chin. J. Cancer Res..

[17] Pongas G.N., Ramos J.C. (2022). HIV-associated lymphomas: Progress and new challenges.. J. Clin. Med..

[18] Schommers P., Hentrich M., Hoffmann C. (2015). Survival of AIDS ‐related diffuse large B‐cell lymphoma, Burkitt lymphoma, and plasmablastic lymphoma in the German HIV Lymphoma Cohort.. Br. J. Haematol..

[19] Noy A. (2019). Optimizing treatment of HIV-associated lymphoma.. Blood.

[20] Wang C., Wu Y., Liu J. (2023). Impact of initial chemotherapy cycles and clinical characteristics on outcomes for HIV-associated diffuse large B cell lymphoma patients: The Central and Western China AIDS Lymphoma League 001 study (CALL-001 study).. Front. Immunol..

[21] Vargas J.C., Marques M.O., Pereira J. (2023). Factors associated with survival in patients with lymphoma and HIV.. AIDS.

[22] Barta S.K., Samuel M.S., Xue X. (2015). Changes in the influence of lymphoma- and HIV-specific factors on outcomes in AIDS-related non-Hodgkin lymphoma.. Ann. Oncol..

[23] Yin H., Qu J., Peng Q., Gan R. (2019). Molecular mechanisms of EBV-driven cell cycle progression and oncogenesis.. Med. Microbiol. Immunol..

[24] Navarro W.H., Kaplan L.D. (2006). AIDS-related lymphoproliferative disease.. Blood.

[25] Yarchoan R., Uldrick T.S. (2018). HIV-associated cancers and related diseases.. N. Engl. J. Med..

[26] Gopal S., Patel M.R., Yanik E.L. (2013). Temporal trends in presentation and survival for HIV-associated lymphoma in the antiretroviral therapy era.. J. Natl. Cancer Inst..

[27] Chapman J.R., Bouska A.C., Zhang W. (2021). EBV‐positive HIV‐associated diffuse large B cell lymphomas are characterized by JAK/STAT (STAT3) pathway mutations and unique clinicopathologic features.. Br. J. Haematol..

[28] Vaughan J., Patel M., Suchard M. (2024). Derangements of immunological proteins in HIV-associated diffuse large B-cell lymphoma: The frequency and prognostic impact.. Front. Cell. Infect. Microbiol..

[29] Vidya Vijayan K.K., Karthigeyan K.P., Tripathi S.P., Hanna L.E. (2017). Pathophysiology of CD4+ T-cell depletion in HIV-1 and HIV-2 infections.. Front. Immunol..

[30] Nie P., Cao Z., Yu R. (2024). Targeting p97–Npl4 interaction inhibits tumor Treg cell development to enhance tumor immunity.. Nat. Immunol..

[31] Wang X.M., Zhang J.Y., Xing X. (2022). Global transcriptomic characterization of T cells in individuals with chronic HIV-1 infection.. Cell Discov..

[32] Perdomo-Celis F., Taborda N.A., Rugeles M.T. (2019). CD8+ T-cell response to hiv infection in the era of antiretroviral therapy.. Front. Immunol..

[33] Dolina J.S., Van Braeckel-Budimir N., Thomas G.D., Salek-Ardakani S. (2021). CD8+ T cell exhaustion in cancer.. Front. Immunol..

[34] Chevalier M.F., Weiss L. (2013). The split personality of regulatory T cells in HIV infection.. Blood.

[35] López-Abente J., Correa-Rocha R., Pion M. (2016). Functional mechanisms of treg in the context of HIV infection and the janus face of immune suppression.. Front. Immunol..

[36] Moreno-Fernandez M.E., Presicce P., Chougnet C.A. (2012). Homeostasis and function of regulatory T cells in HIV/SIV infection.. J. Virol..

[37] Swaminathan S., Scorza T., Yero A. (2023). Impact of in vitro HIV infection on human thymic regulatory T cell differentiation.. Front. Microbiol..

[38] Yero A., Bouassa R.S.M., Ancuta P., Estaquier J., Jenabian M.A. (2023). Immuno-metabolic control of the balance between Th17-polarized and regulatory T-cells during HIV infection.. Cytokine Growth Factor Rev..

[39] Deeks S.G. (2011). HIV infection, inflammation, immunosenescence, and aging.. Annu. Rev. Med..

[40] Chauvin M., Sauce D. (2022). Mechanisms of immune aging in HIV.. Clin. Sci..

[41] Chan J.Y., Somasundaram N., Grigoropoulos N. (2023). Evolving therapeutic landscape of diffuse large B-cell lymphoma: Challenges and aspirations.. Discov. Oncol..

[42] Liu Z., Liang Q., Ren Y. (2023). Immunosenescence: Molecular mechanisms and diseases.. Signal Transduct. Target. Ther..

[43] Di M., Huntington S.F., Olszewski A.J. (2021). Challenges and opportunities in the management of diffuse large B-cell lymphoma in older patients.. Oncologist.

[44] Huguet M., Navarro J.T., Moltó J., Ribera J.M., Tapia G. (2023). Diffuse large B-cell lymphoma in the HIV setting.. Cancers.

[45] Hocqueloux L., Avettand-Fènoël V., Jacquot S. (2013). Long-term antiretroviral therapy initiated during primary HIV-1 infection is key to achieving both low HIV reservoirs and normal T cell counts.. J. Antimicrob. Chemother..

[46] Jain V., Hartogensis W., Bacchetti P. (2013). Antiretroviral therapy initiated within 6 months of HIV infection is associated with lower T-cell activation and smaller HIV reservoir size.. J. Infect. Dis..

[47] Chun T.W., Justement J.S., Pandya P. (2002). Relationship between the size of the human immunodeficiency virus type 1 (HIV-1) reservoir in peripheral blood CD4+ T cells and CD4+:CD8+ T cell ratios in aviremic HIV-1-infected individuals receiving long-term highly active antiretroviral therapy.. J. Infect. Dis..

[48] Ruppert A.S., Dixon J.G., Salles G. (2020). International prognostic indices in diffuse large B-cell lymphoma: A comparison of IPI, R-IPI, and NCCN-IPI.. Blood.

[49] Sehn L.H., Salles G. (2021). Diffuse large B-cell lymphoma.. N. Engl. J. Med..

[50] Scott D.W., Mottok A., Ennishi D. (2015). Prognostic significance of diffuse large b-cell lymphoma cell of origin determined by digital gene expression in formalin-fixed paraffin-embedded tissue biopsies.. J. Clin. Oncol..

