# Supplementary material for: The Characteristics of Peripheral Blood Lymphocyte Subsets in HIV-related Diffuse Large B-cell Lymphoma Patients and Their Impact on Treatment Efficacy
Source: Curr HIV Res. 2025 Aug 26;24(1):62–73. doi: 10.2174/011570162X369231250818043532 (PMC13555762; doi:10.2174/011570162X369231250818043532)
Supplement: Supplementary file 1 [file CHIVR-24-1-62_SD1.pdf]

Supplementary Material

The Characteristics of Peripheral Blood Lymphocyte Subsets in HIV-related Diffuse Large B-cell Lymphoma Patients and Their Impact on Treatment Efficacy

Huiyu Xiang[1,2,#], Can Lin[2,#], Shuang Chen[2], Yu Peng[2], Tingting Jiang[2], Changhai Lin[3,4,] Qing Xiao[2], Xiaomei Zhang[2], Tingting Liu[2], Nanjun Li[2], Xinyi Tang[1,2], Yakun Zhang[1,2], Junxi Liu[1,2] and Zailin Yang[2,*]

[1]School of Medicine, Chongqing University, Chongqing, 400030, China; [2]Department of Hematology-Oncology, Chongqing Key Laboratory for the Mechanism and Intervention of Cancer Metastasis, Chongqing University Cancer Hospital, Chongqing, 400030, China; [3]Department of Laboratory Medicine, Chongqing University Cancer Hospital, Chongqing, China; [4]School of Life Sciences, Chongqing University, Chongqing, China

Supplementary Table 1. Baseline demographics and disease characteristics [Median (Q1, Q3)].

P	t/Z	HIV-associated DLBCL n=51	Healthy donors n=42	P	t/Z	DLBCL n=50	Healthy donors n=42	P	t/Z
0.891	0.137	749.00(543.00, 1178.00)	1235.50(1035.75,1621.25)	<0.001	####	760.00(612.50, 1094.75)	1235.50(1035.75,1621.25)	<0.001	####
<0.001	4.847	565.00(294.00, 821.00)	419.50(305.00, 564.50)	0.109	1.604	243.00(162.50, 401.75)	419.50(305.00, 564.50)	0.003	####
<0.001	-5.602	182.00(107.00,261.00)	691.50(524.75, 897.25)	0.000	####	434.00(305.50, 566.50)	691.50(524.75, 897.25)	<0.001	####
0.985	-0.018	123.00(45.00, 217.00)	209.00(132.75, 314.75)	<0.001	####	120.50(46.75, 194.75)	209.00(132.75, 314.75)	<0.001	####
0.186	-1.323	133.00(81.00, 217.00)	290.50(181.25, 521.00)	<0.001	####	156.50(92.00, 265.75)	290.50(181.25, 521.00)	<0.001	####
0.017	-2.383	4.50(2.00, 7.25)	8.00(3.75, 14.00)	0.004	####	8.00(4.00, 11.00)	8.00(3.75, 14.00)	0.509	####
0.221	1.232	74.50(64.36，80.95)	68.36(63.04,75.07)	0.020	2.368	69.77(62.78,76.36)	68.36(63.04,75.07)	0.280	1.087
<0.001	7.328	52.41(41.92,59.50)	22.78(16.92,28.22)	<0.001	8.103	23.34(17.76,32.76)	22.78(16.92,28.22)	0.272	1.098
<0.001	-7.798	15.47(11.20, 22.16)	37.78(30.10,43.44)	<0.001	####	36.94(32.41,43.22)	37.78(30.10,43.44)	0.932	####
0.729	-0.346	10.23(5.90,15.70)	10.58(8.04 ,13.28)	0.487	####	12.23(4.53, 15.98)	10.58(8.04 ,13.28)	0.913	####
0.264	-1.117	10.50(7.12, 22.34)	16.66(12.05, 24.72)	0.019	####	15.41(8.8, 25.04)	16.66(12.05, 24.72)	0.271	####
<0.001	3.647	3.19(1.98, 4.49)	1.14 (0.63 ,1.88)	<0.001	5.503	1.68(1.09, 2.26)	1.14 (0.63 ,1.88)	0.019	2.349

Supplementary Table 2. Comparison of peripheral blood lymphocyte subset levels in DLBCL, HIV-DLBCL, and HD at various ages [Median (Q1, Q3)]

-	≥60 years (n=46)											
	**HIV-DLBCL n=11**	**DLBCL n=30**	**P**	**t/Z**	**HIV-DLBCL n=11**	**Healthy donors n=5**	**P**	**t/Z**	**DLBCL n=30**	**Healthy donors n=5**	**P**	**t/Z**
$CD3^+$ (cells/μL)	543.00(304.00,746.00)	724.00(599.75,974.00)	0.181	-1.363	543.00(304.00,746.00)	1127.90 (699.33 ,1710.59)	0.033	-2.364	724.00(599.75,974.00)	1127.90 (699.33 ,1710.59)	0.043	-2.102
$CD8^+$ (cells/μL)	440.00(157.00,596.00)	215.00(151.75,386.25)	0.165	-1.412	440.00(157.00,596.00)	491.46(231.79,631.62)	0.583	-0.623	215.00(151.75,386.25)	491.46(231.79,631.62)	0.095	-1.697
$CD4^+$ (cells/μL)	146.00(.49.00,191.00)	406.50(311.25,505.25)	<0.001	-4.116	146.00(.49.00,191.00)	525.20(422.48,817.43)	<0.001	-3.096	406.50(311.25,505.25)	525.20(422.48,817.43)	0.175	-1.357
$CD19^+$ (cells/μL)	49.00(28.00,159.00)	103.50(39.25,164.25)	0.391	-0.868	49.00(28.00,159.00)	216.85(96.24,257.01)	0.052	-1.984	103.50(39.25,164.25)	216.85(96.24,257.01)	0.155	-1.462
$CD56^+$ $CD16^+$(cells/μL)	136.00(91.00,274.00)	156.50(91.75,265.75)	0.805	-0.265	136.00(91.00,274.00)	175.87(169.68,603.55)	0.145	-1.529	156.50(91.75,265.75)	175.87(169.68,603.55)	0.219	-1.273
Treg (cells/μL)	4.53(1.19,7.78)	6.00(4.00,10.25)	0.219	-1.276	4.53(1.19,7.78)	8.10(2.82,11.95)	0.310	-1.149	6.00(4.00,10.25)	8.10(2.82,11.95)	0.837	-0.236
$CD3^+$ %	65.38(63.60,74.50)	68.25(60.75,74.88)	0.595	-0.536	65.38(63.60,74.50)	67.88(62.83,75.22)	0.716	-0.371	68.25(60.75,74.88)	71.38(59.30,76.57)	0.995	0.007
$CD8^+$ %	52.84(34.40,57.89)	23.31(16.63,31.39)	<0.001	-3.396	52.84(34.40,57.89)	22.65(16.61,28.22)	0.002	-2.889	23.31(16.63,31.39)	24.58(21.67,28.34)	0.732	-0.377
$CD4^+$ %	14.50(8.51,23.00)	36.31(31.41,41.51)	<0.001	-4.384	14.50(8.51,23.00)	37.42(30.32,43.53)	0.004	-2.855	36.31(31.41,41.51)	40.80(26.30,43.65)	0.992	0.01
$CD19^+$ %	9.93(5.77,15.79)	10.31(4.53,16.37)	0.591	-0.559	9.93(5.77,15.79)	10.83(8.58,14.05)	0.743	-0.397	10.31(4.53,16.37)	10.10(8.95,817.43)	0.945	-0.094
$CD56^+$ $CD16^+$ %	22.34(9.30,31.00)	18.65(8.93,25.35)	0.315	-1.03	22.34(9.30,31.00)	16.91(11.75,24.74)	0.827	-0.283	18.65(8.93,25.35)	16.41(10.67,28.85)	0.982	-0.047
Treg%	3.28(2.24,13.66)	1.77(0.96,2.29)	0.017	2.388	3.28(2.24,13.66)	1.14(0.63,1.82)	0.032	2.151	1.77(0.96,2.29)	1.75(0.44,2.44)	0.669	0.428

<60years(n=97)											
**HIV-DLBCL n=40**	**DLBCL n=20**	**P**	**t/Z**	**HIV-DLBCL n=40**	**Healthydonors n=37**	**P**	**t/Z**	**DLBCL n=20**	**Healthy donors n=37**	**P**	**t/Z**
830.00(664.00,1231.25)	816.00(618.25,1204.00)	0.982	0.023	830.00(664.00,1231.25)	1250.53(1049.61,1621.68)	<0.001	-3.394	816.00(618.25,1204.00)	1250.53(1049.61,1621.68)	0.005	-2.812
585.00(415.50,909.75)	268.00(169.75,461.00)	<0.001	3.782	585.00(415.50,909.75)	418.22(311.41,542.40)	0.011	2.548	268.00(169.75,461.00)	418.22(311.41,542.40)	0.102	-1.638
193.00(111.25,270.25)	488.00(289.50,769.25)	<0.001	-3.907	193.00(111.25,270.25)	695.84(526.28,897.31)	<0.001	-7.485	488.00(289.50,769.25)	695.84(526.28,897.31)	0.022	-2.296
129.00(47.50,248.25)	132.50(50.00,256.00)	0.820	-0.227	129.00(47.50,248.25)	209.01(136.35,327.84)	0.003	-2.936	132.50(50.00,256.00)	209.01(136.35,327.84)	0.029	-2.189
131.50(80.25,204.75)	157.50(102.00,288.50)	0.370	-0.897	131.50(80.25,204.75)	339.74(194.60,546.67)	<0.001	-5.293	157.50(102.00,288.50)	339.74(194.60,546.67)	<0.001	-3.465
4.18(2.40,7.48)	9.00(4.25,12.50)	0.031	-2.156	4.18(2.40,7.48)	8.04(3.68,14.39)	0.013	-2.495	9.00(4.25,12.50)	8.04(3.68,14.39)	0.977	-0.028
75.80(65.32,82.00)	74.77(64.20,78.74)	0.458	0.746	75.80(65.32,82.00)	67.88(62.83,75.22)	0.004	2.978	74.77(64.20,78.74)	67.88(62.83,75.22)	0.096	1.692
52.36(43.98,61.13)	23.34(19.33,33.01)	<0.001	5.167	52.36(43.98,61.13)	22.65(16.61,28.22)	<0.001	7.632	23.34(19.33,33.01)	22.65(16.61,28.22)	0.240	1.174
15.49(12.16,21.47)	38.07(33.54,43.97)	<0.001	-5.618	15.49(12.16,21.47)	37.42(30.32,43.53)	<0.001	-6.819	38.07(33.54,43.97)	37.42(30.32,43.53)	0.952	-0.061
10.54(6.46,15.59)	13.22(4.08,15.88)	0.672	-0.423	10.54(6.46,15.59)	10.83(8.58,14.05)	0.625	-0.489	13.22(4.08,15.88)	10.83(8.58,14.05)	0.604	-0.518
9.39(6.89,17.77)	12.99(7.73,22.90)	0.357	-0.921	9.39(6.89,17.77)	16.91(11.75,24.74)	0.002	-3.037	12.99(7.73,22.90)	16.91(11.75,24.74)	0.113	-1.587
3.18(1.92,4.53)	1.50(1.33,2.30)	0.027	2.209	3.18(1.92,4.53)	1.14(0.63,1.82)	<0.001	4.845	1.50(1.33,2.30)	1.14(0.63,1.82)	0.022	2.283

**HIV-DLBCL (n=51)**				**DLBCL (n=50)**			
**≥ 60 years (n=11)**	**<60 years(n=40)**	**P**	**t/Z**	**≥ 60 years (n=30)**	**<60 years(n=20)**	**P**	**t/Z**
543.00(304.00,746.00)	830.00(664.00,1231.25)	0.056	-1.958	724.00(599.75,974.00)	816.00(618.25,1204.00)	0.209	-1.274
440.00(157.00,596.00)	585.00(415.50,909.75)	0.028	-1.924	215.00(151.75,386.25)	268.00(169.75,461.00)	0.171	-0.96
146.00(.49.00,191.00)	193.00(111.25,270.25)	0.065	-1.523	406.50(311.25,505.25)	488.00(289.50,769.25)	0.238	-0.723
49.00(28.00,159.00)	129.00(47.50,248.25)	0.073	-1.466	103.50(39.25,164.25)	132.50(50.00,256.00)	0.241	-0.713
136.00(91.00,274.00)	131.50(80.25,204.75)	0.300	-0.538	156.50(91.75,265.75)	157.50(102.00,288.50)	0.467	-0.089
4.53(1.19,7.78)	4.18(2.40,7.48)	0.456	-0.130	6.00(4.00,10.25)	9.00(4.25,12.50)	0.129	-1.143
65.38(63.60,74.50)	75.80(65.32,82.00)	0.033	-2.197	68.25(60.75,74.88)	74.77(64.20,78.74)	0.265	-1.128
52.84(34.40,57.89)	52.36(43.98,61.13)	0.231	-1.213	23.31(16.63,31.39)	23.34(19.33,33.01)	0.719	-0.361
14.50(8.51,23.00)	15.49(12.16,21.47)	0.387	-0.298	36.31(31.41,41.51)	38.07(33.54,43.97)	0.308	-0.515
9.93(5.77,15.79)	10.54(6.46,15.59)	0.535	-0.625	10.31(4.53,16.37)	13.22(4.08,15.88)	0.337	-0.436
22.34(9.30,31.00)	9.39(6.89,17.77)	0.016	-2.141	18.65(8.93,25.35)	12.99(7.73,22.90)	0.136	-1.109
3.28(2.24,13.66)	3.18(1.92,4.53)	0.307	-0.553	1.77(0.96,2.29)	1.50(1.33,2.30)	0.346	-0.406

**Healthy donors (n=42)**			
**≥ 60 years (n=5)**	**<60 years(n=37)**	**P**	**t/Z**
1127.90 (699.33 ,1710.59)	1250.53(1049.61,1621.68)	0.260	-0.680
491.46(231.79,631.62)	418.22(311.41,542.40)	0.425	-0.214
525.20(422.48,817.43)	695.84(526.28,897.31)	0.152	-1.068
216.85(96.24,257.01)	209.01(136.35,327.84)	0.260	-0.68
175.87(169.68,603.55)	339.74(194.60,546.67)	0.236	-0.757
8.10(2.82,11.95)	8.04(3.68,14.39)	0.325	-0.485
67.88(62.83,75.22)	67.88(62.83,75.22)	0.827	0.220
22.65(16.61,28.22)	22.65(16.61,28.22)	0.386	0.876
37.42(30.32,43.53)	37.42(30.32,43.53)	0.760	-0.308
10.83(8.58,14.05)	10.83(8.58,14.05)	0.558	-0.591
16.91(11.75,24.74)	16.91(11.75,24.74)	0.982	-0.023
1.14(0.63,1.82)	1.14(0.63,1.82)	0.550	0.602

Supplementary Table 3. Comparison of peripheral blood lymphocyte subset levels across different treatment response groups after four cycles of chemotherapy. [Median (Q1, Q3)].

-	HIV-DLBCL (n=27)			
	**AchievementofCR (n=11)**	**CRnotmet (n=16)**	**P**	**t/Z**
$CD3^+$ (cells/μL)	1117.00(746.00,1241.00)	762.50(546.75,1093.00)	0.133	-1.135
$CD8^+$ (cells/μL)	725.00(513.00,810.00)	527.00(324.00,813.25)	0.147	-1.086
$CD4^+$ (cells/μL)	244.00(146.00,360.00)	188.50(101.00,266.25)	0.208	0.839
$CD19^+$ (cells/μL)	159.00(46.00,184.00)	153.50(56.25,291.00)	0.417	-0.182
$CD56^+$ $CD16^+$(cells/μL)	134.00(91.00,238.00)	140.50(95.00,231.50)	0.214	-0.815
Treg (cells/μL)	4.35(3.30,5.62)	2.80(1.36,4.00)	0.298	-0.586
$CD3^+$ %	76.33(63.40,80.61)	68.93(61.73,78.22)	0.048	-1.678
$CD8^+$ %	49.81(41.92,59.50)	48.59(37.20,60.14)	0.719	0.364
$CD4^+$ %	19.13(14.50,25.20)	14.53(8.77,22.10)	0.459	0.751
$CD19^+$ %	10.80(3.42,15.11)	13.90(10.60,17.18)	0.307	-0.524
$CD56^+CD16^+$ %	9.30(7.50,25.30)	15.24(6.50,21.90)	0.074	-1.480
Treg%	2.88(1.30,4.14)	2.78(2.05,5.03)	0.909	-0.116

DLBCL (n=32)			
**AchievementofCR (n=15)**	**CRnotmet (n=17)**	**P**	**t/Z**
718.00(321.00,1144.00)	801.00(658.50,1143.00)	0.272	-0.623
219.00(154.00,509.00)	269.00(182.00,556.50)	0.369	-0.359
379.00(187.00,506.00)	391.00(302.00,714.50)	0.430	-0.189
95.00(12.00,129.00)	134.00(51.00,183.50)	0.464	-0.112
127.00(88.00,258.00)	182.00(96.50,321.00)	0.156	-1.038
4.00(2.00,8.00)	9.00(4.00,12.00)	0.400	-0.267
70.22(64.33,79.47)	73.04(62.47,78.77)	0.832	-0.215
29.89(18.03,35.89)	23.84(21.34,34.68)	0.407	0.848
35.13(29.78,38.66)	36.78(29.76,43.47)	0.581	-0.560
8.13(4.62,15.01)	12.57(4.76,15.64)	0.299	-0.551
19.62(8.88,25.62)	17.39(6.71,23.00)	0.485	-0.057
1.62(0.80,2.10)	1.72(1.21,2.90)	0.327	-0.472

AchievementofCR (n=26)				CR not met (n=33)			
HIV-DLBCL (n=11)	DLBCL (n=15)	P	t/Z	HIV-DLBCL (n=16)	DLBCL (n=17)	P	t/Z
1117.00(746.00,1241.00)	718.00(321.00,1144.00)	0.357	-0.388	762.50(546.75,1093.00)	801.00(658.50,1143.00)	0.049	-1.671
725.00(513.00,810.00)	219.00(154.00,509.00)	0.495	-0.028	527.00(324.00,813.25)	269.00(182.00,556.50)	0.463	-0.103
244.00(146.00,360.00)	379.00(187.00,506.00)	0.127	-1.165	188.50(101.00,266.25)	391.00(302.00,714.50)	0.003	-2.669
159.00(46.00,184.00)	95.00(12.00,129.00)	0.198	-0.913	153.50(56.25,291.00)	134.00(51.00,183.50)	0.202	-0.979
134.00(91.00,238.00)	127.00(88.00,258.00)	0.162	-1.446	140.50(95.00,231.50)	182.00(96.50,321.00)	0.042	-1.740
4.35(3.30,5.62)	4.00(2.00,8.00)	0.478	-0.101	2.80(1.36,4.00)	9.00(4.00,12.00)	0.021	-2.032
76.33(63.40,80.61)	70.22(64.33,79.47)	0.210	-0.832	68.93(61.73,78.22)	73.04(62.47,78.77)	0.486	-0.052
49.81(41.92,59.50)	29.89(18.03,35.89)	0.293	1.076	48.59(37.20,60.14)	23.84(21.34,34.68)	0.002	3.417
19.13(14.50,25.20)	35.13(29.78,38.66)	0.225	-1.248	14.53(8.77,22.10)	36.78(29.76,43.47)	<0.001	-4.296
10.80(3.42,15.11)	8.13(4.62,15.01)	0.027	-2.055	13.90(10.60,17.18)	12.57(4.76,15.64)	0.053	-1.627
9.30(7.50,25.30)	19.62(8.88,25.62)	0.284	-0.610	15.24(6.50,21.90)	17.39(6.71,23.00)	0.473	-0.086
2.88(1.30,4.14)	1.62(0.80,2.10)	0.028	2.409	2.78(2.05,5.03)	1.72(1.21,2.90)	0.009	-2.345

Supplementary Table 4. Comparison of peripheral blood lymphocyte subsets between ART-treated and ART-naive HIV-DLBCL patients [Median (Q1, Q3)].

-	Antiviral Treatment Group (n=32)	No antiviral Treatment Group (n=19)	P	t/Z
$CD3^+$ (cells/μL)	762.50 (543.25, 1288.25)	743.00(535.00,1140.00)	0.276	-0.604
$CD8^+$ (cells/μL)	581.50 (288.00, 915.50)	546.00(440.00,810.00)	0.397	-0.273
$CD4^+$ (cells/μL)	188.50(128.00,270.25)	161.00(55.00,210.00)	0.101	1.673
$CD19^+$ (cells/μL)	141.00 (48.25, 249.25)	70.00 (27.00, 184.00)	0.091	-1.344
$CD56^+$ $CD16^+$(cells/μL)	142.50(91.25,226.75)	108.00 (61.00, 142.00)	0.134	-1.12
Treg (cells/μL)	5.00(3.00,8.00)	3.00(1.00,7.00)	0.061	-1.564
$CD3^+$ %	72.60(64.24,80.61)	75.79(64.40,82.00)	0.825	-0.222
$CD8^+$ %	50.39(38.35,57.93)	52.84(47.62,60.90)	0.224	-1.233
$CD4^+$ %	15.95 (12.16, 22.89)	15.23(8.80,17.77)	0.154	-1.031
$CD19^+$ %	11.59(6.54,16.15)	7.28(5.40,13.10)	0.121	1.577
$CD56^+CD16^+$ %	10.50 (7.05, 19.05)	10.50 (7.50, 23.50)	0.403	-0.253
Treg%	3.28 (2.29, 4.67)	2.78(1.49,4.55)	0.262	-1.165
